# Estimating the incidence of interstitial lung diseases in the Cree of Eeyou Istchee, northern Québec

**DOI:** 10.1371/journal.pone.0184548

**Published:** 2017-09-08

**Authors:** Matthieu Storme, Alexandre Semionov, Deborah Assayag, Michael Lefson, Darlene Kitty, David Dannenbaum, Jill Torrie, Pierre Lejeune, Elizabeth Robinson, Faiz Ahmad Khan

**Affiliations:** 1 Division of Respiratory Medicine, McGill University, Montreal, Quebec, Canada; 2 Radiology Dept., McGill University Health Centre, Montreal, Quebec, Canada; 3 Pulmonary Division, Jewish General Hospital, Montreal, Quebec, Canada; 4 Department of Medicine, Cree Board of Health and Social Services of the James Bay, Montreal, Quebec, Canada; 5 Department of Public Health, Cree Board of Health and Social Services of the James Bay, Montreal, Quebec, Canada; 6 Department of Epidemiology, Biostatistics and Occupational Health, McGill University, Montreal, Quebec, Canada; 7 Montreal Chest Institute, McGill University Health Centre, Montreal, Quebec, Canada; 8 Respiratory Epidemiology & Clinical Research Unit, Centre for Outcomes Research and Evaluation, Research Institute of the McGill University Health Centre, Montreal, Quebec, Canada; University of Alabama at Birmingham, UNITED STATES

## Abstract

**Background:**

Little is known about the epidemiology of interstitial lung disease (ILD) amongst Canada’s Indigenous populations. Clinicians working in Eeyou Istchee (the Cree territory of the James Bay region of Québec, population 17, 956) suspected that ILD was more common in this area. We sought to identify all prevalent and incident cases of ILD in Eeyou Istchee between 2006 and 2013, to describe characteristics of affected patients, distribution of subtypes, and estimate disease incidence.

**Methods:**

Potential ILD cases amongst Eeyou Istchee residents were identified by searching hospitalization databases and lists of patients on long term home oxygen in the region’s nine communities, and surveying physicians and nurses. Clinical, radiological and pathological data were reviewed. Potential cases were classified as ‘Definite ILD’ if an open lung biopsy demonstrated ILD or, in the absence of histopathologic confirmation, if their thoracic CT imaging was deemed consistent with ILD by a panel of two respirologists and a chest radiologist. Potential cases for whom CT images could not be retrieved for our review were not eligible for classification as Definite ILD, unless they had undergone open lung biopsy. The Definite ILD group was further categorized by subtype of ILD. For usual interstitial pneumonia and non-specific interstitial pneumonitis patterns, we assumed cases were idiopathic in the absence of documentation of connective tissue disease or occupational exposures in the medical chart. For Definite ILD and the most common subtype, we calculated the average annual incidence rates, age-standardized to the province of Quebec, for 2006 to 2013, using a gamma distribution to calculate 95% confidence intervals.

**Results:**

Of 167 potential cases, 52 were categorized as Definite ILD: 14 on the basis of histopathology and 38 on the basis of CT imaging alone. Six patients had a prior history of connective tissue disease. Information on occupation was recorded in the charts of 18/52 (35%) cases, and missing in the remainder. We found the most common subtype was idiopathic pulmonary fibrosis (27/52, 52%), followed by idiopathic non-specific interstitial pneumonia (13/52, 25%), and secondary usual interstitial pneumonia associated with connective tissue diseases (5/52, 10%). The age-standardized annual incidence between 2006–2013 was 80 per 100,000 person-years observed (PYO) for ILD, and 46 per 100,000 PYO for idiopathic pulmonary fibrosis.

**Interpretation:**

The incidence of ILD and of idiopathic pulmonary fibrosis in Eeyou Istchee may be higher than rates reported in other populations; however, cautious interpretation is required due to the lack of histopathological confirmation in the majority of cases, and our reliance on chart review to exclude secondary causes. A prospective study of incident cases with standardized assessments to establish the types of ILD and to assess for potential causes could overcome some of the limitations of the present analysis. Studies evaluating ILD incidence and subtype distribution in other Indigenous populations would also be of interest.

## Introduction

A number of disparities in respiratory health exist between Canada’s Indigenous populations and their non-Indigenous counterparts, with the former having higher rates of cigarette smoking, [1] tuberculosis, [2] self-reported asthma, [3] and chronic obstructive pulmonary disease. [4] No data exist on the occurrence of interstitial lung disease (ILD) in Indigenous groups.

ILD is a term for a group of disorders characterized by fibrotic and inflammatory changes in the interstitial tissue of the lung. [5–7] Idiopathic pulmonary fibrosis (IPF), the most common subtype of ILD, [8,9] is diagnosed based on a histologic or radiologic pattern of usual interstitial pneumonia and the absence of conditions or exposures known to cause this pattern of lung injury.[7,10] IPF has a poor prognosis, with a median survival of approximately four years following diagnosis.[11]

We undertook an epidemiologic investigation of ILD amongst the Cree of Eeyou Istchee, one of Quebec’s Indigenous peoples. The study was motivated by family physicians and public health officials who suspected that the number of patients with ILD in this region was disproportionately elevated relative to the size of the population. Our objectives were to identify all residents of Eeyou Istchee that were diagnosed with ILD, in order to determine the distribution of ILD subtypes, describe patient characteristics, and estimate incidence of ILD overall and of the most common subtype. A secondary objective was to estimate survival of ILD cases.

## Methods

### Setting

**Fig 1:**
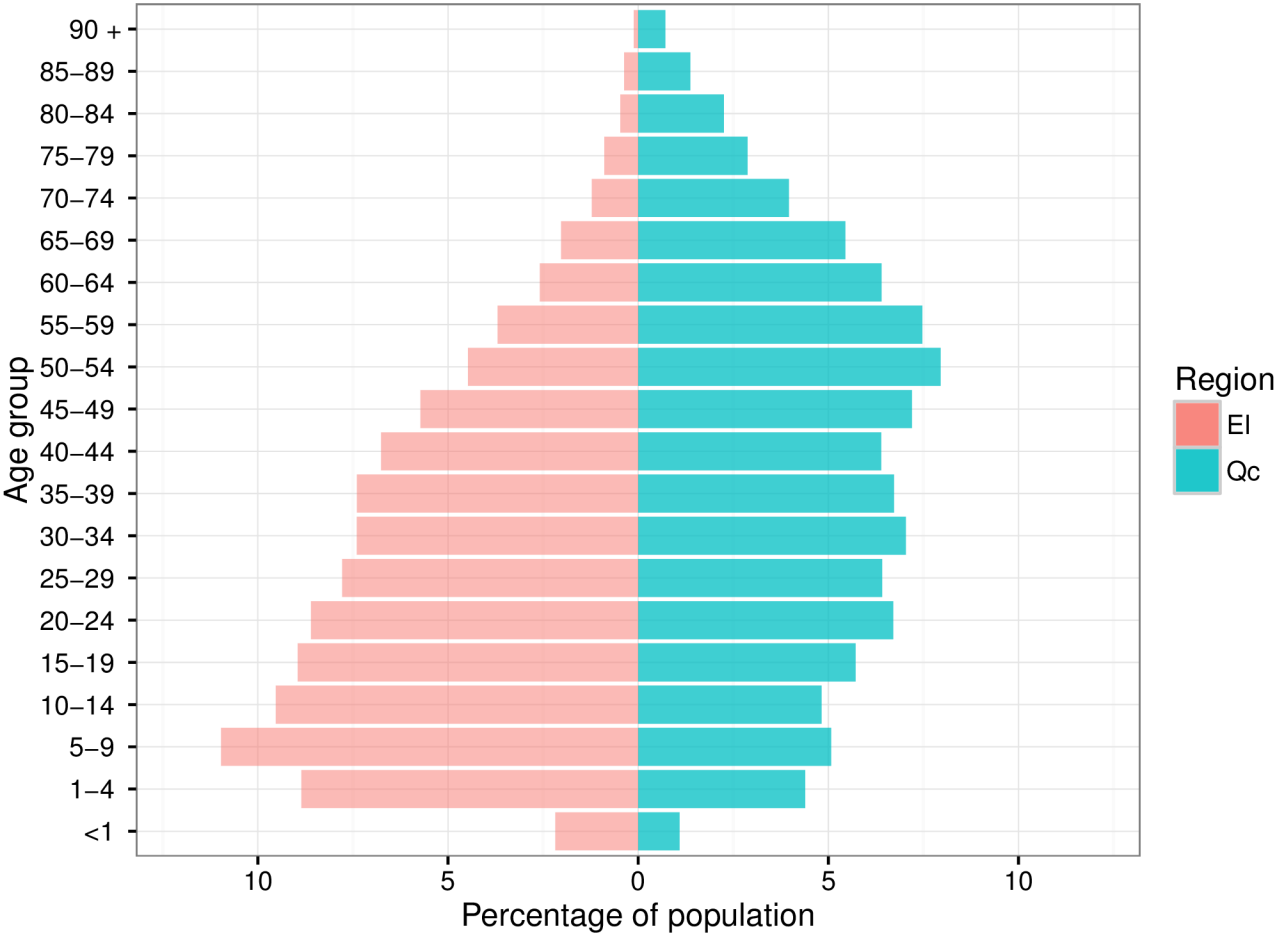
Comparison of age distribution of populations of Eeyou Istchee (pink) and province of Québec (green). EI: Eeyou Istchee; Qc: Québec. Population pyramids were created using package ggplot2 in R statistical software and data from Ministère de la Santé et des Services Sociaux. Institut de la statistique du Québec Population Estimates and Projections, 1996 to 2036, March 2015 (based on Statistics Canada 2011 census).

Eeyou Istchee is the traditional name of the Cree territory of the James Bay region in northern Québec. Over 95% of Eeyou Istchee’s 17,956 residents are Cree.[12] This region’s population is much younger compared to the rest of the province of Québec (Fig 1). Each of the region’s nine communities has a local clinic that provides primary health care services. Patients requiring hospitalization are transferred either to the regional hospital in Chisasibi, or to one of three referral centers outside of Eeyou Istchee: Val d’Or Hospital, Chibougamau Health Centre, and the McGill University Health Centre.

### Study design

We identified all prevalent cases of ILD amongst Eeyou Istchee residents and estimated the incidence for the period from January 1^st^, 2006 and December 31^st^, 2013. To identify persons with ILD, we first identified the region’s residents with a diagnosis of ILD mentioned in their medical records, whom we called “potential cases”. Next, we determined in which of these potential cases the diagnosis of ILD was strongly supported by radiological or pathological data, a group we called “Definite ILD”. The Definite ILD group was further categorized by their ILD subtype. The number of Definite ILD cases diagnosed after January 1^st^ 2006 was used to calculate incidence. Details on how we identified potential cases, determined which had Definite ILD, assigned subtypes of ILD, and calculated incidence, are described below.

Potential cases of ILD were identified by three methods. (1) We searched the four hospitals that serve the Eeyou Istchee region for persons that had resided in the Quebec health ministry’s administrative region 18, which is the Eeyou Istchee region, and had been hospitalized with a discharge diagnosis that included ILD ICD-10 codes (see S1 Table). (2) We reviewed lists of patients on long-term home oxygen treatment in each community’s primary care clinic. These lists were used because these clinics do not have databases that enumerate the diagnoses of patients in the villages, but they do have lists of people that receive home oxygen. We collected the names and dates of births of patients on these lists and searched their clinic chart as well as their hospital records to see if an ILD had been mentioned. (3) We contacted healthcare workers to ask them to provide the names and other identifying information of patients with ILD, and we then searched and extracted data from the medical records of these patients. The healthcare workers were identified through an email listserv. A detailed data extraction (described below) was performed for potential cases that met the following criteria: the person had not died prior to January 1^st^ 2006, a medical chart was available, and an ILD diagnosis was mentioned somewhere in the medical chart.

For the detailed data extraction, we used a standard form to collect information on clinical presentation, medications, exposure to radiation therapy, laboratory and pulmonary function tests, as well as lung biopsy and bronchoscopy reports, and dates and causes of death. We obtained all available thoracic computed tomography (CT) scan imaging.

All CT scan images were independently reviewed by a respirology team (consisting of a respirology resident (MS) and a respirologist (FAK)), and a chest radiologist (AS). Readings were performed blind to the clinical data. For each potential case, CT-imaging was classified as either “consistent with ILD,” “equivocal for ILD”, or “not consistent with ILD”: the two members of the respirology team reviewed each scan together and assigned their categorizations of ILD, and an independent review and categorization was performed by the chest radiologist; differences in readings were then settled by consensus in a face-to-face meeting where all images were reviewed. The weighted kappa statistic for agreement between the Respirology and Radiology interpretation was 0.71 (95%CI: 0.61–0.82), in keeping with the interobserver variability reported in other studies.[13] At the consensus meeting, we further classified CT images that were “Consistent with ILD” by the subtype of ILD pattern. To do so, we first grouped persons based on American Thoracic Society criteria for the usual interstitial pneumonia pattern[14]: definite, possible, or not present. For patients in whom the usual interstitial pneumonia pattern was not present, the most likely subtype of ILD was based on consensus interpretation of CT imaging.

After the radiology consensus had been assigned, the Respirology team (MS, FAK) incorporated clinical data obtained from the chart review for further classification. The first step was to integrate pathology findings for patients where open lung biopsy had been done. Potential cases were classified as “Definite ILD” if an open lung biopsy demonstrated an ILD or, in the absence of a biopsy, if their consensus CT imaging category was Consistent with ILD. For the subtype of ILD, classification was based on histopathology when available, and otherwise on the consensus interpretation of the CT imaging ILD pattern.The next step in integrating clinical information was use of data from the chart review to identify potential causes of ILD. Cases were assumed to be idiopathic in the absence of a past medical history of connective tissues diseases (rheumatoid arthritis, scleroderma, Sjogren’s, lupus, mixed connective tissue disease), and if no occupation known to increase the risk of pneumoconiosis was noted in the chart; for patients in whom such occupations were recorded, we assumed the ILD was idiopathic unless the CT findings were typical for the pneumoconiosis for which their occupation placed them at risk of. Persons with definite or possible usual interstitial pneumonia pattern were classified as IPF if no secondary cause was identified.

### Patient characteristics and survival

For all Definite ILD cases (prevalent and incident), sex, year and age of diagnosis, smoking status, vital status, and results of pulmonary function tests were summarized as proportions or median values with interquartile ranges (IQR). We used the Kaplan-Meier method to estimate median survival from the date of diagnosis which we defined as the earliest date amongst the following: open lung biopsy demonstrating ILD or first CT scan showing abnormalities consistent with ILD. For survival calculations, patients were followed until the date they died, or if their vital status was not known, they were censored at the date of the most recent test included in our data collection.

### Calculation of incidence rates

In order to calculate incidence rates for Definite ILD, and the most common subtype, from January 1^st^ 2006 to December 31^st^ 2013, we excluded patients diagnosed before 2006. To calculate the crude incidence, we divided the number of incident cases by the total PYO in the James Bay Cree Territory from 2006 to 2013 as estimated by the Institut de la Statistique du Québec for the Ministère de la Santé et des Services Sociaux du Québec.[12] Because the Eeyou Istchee population is much younger than that of Québec (Fig 1) while ILD mostly affect older age groups, we calculated age-adjusted incidence rates by stratifying incident cases into strata of age of diagnosis, calculating age-specific rates in the Eeyou Istchee population, and then performed direct standardization of these rates using population data for the province of Quebec from the years 2006–2013.[12] All analyses were performed in SAS, v 9.4. We used a gamma-distribution to calculate 95% confidence intervals for the standardized rates, as proposed by Fay and Feuer.[15]

### Ethical approval

Ethical approval for the study was given by the Institutional Review Board of the McGill University Faculty of Medicine and community review by the Cree Board of Health and Social Services. The need for informed consent was waived given the retrospective nature of the study. Additionally, we obtained approval from the Directors of medical Professional Services at the hospitals where data were collected (Chisasibi, McGill University Health Centre, Val d’Or, and Chibougamau).

## Results

### Identification of potential cases

**Fig 2:**
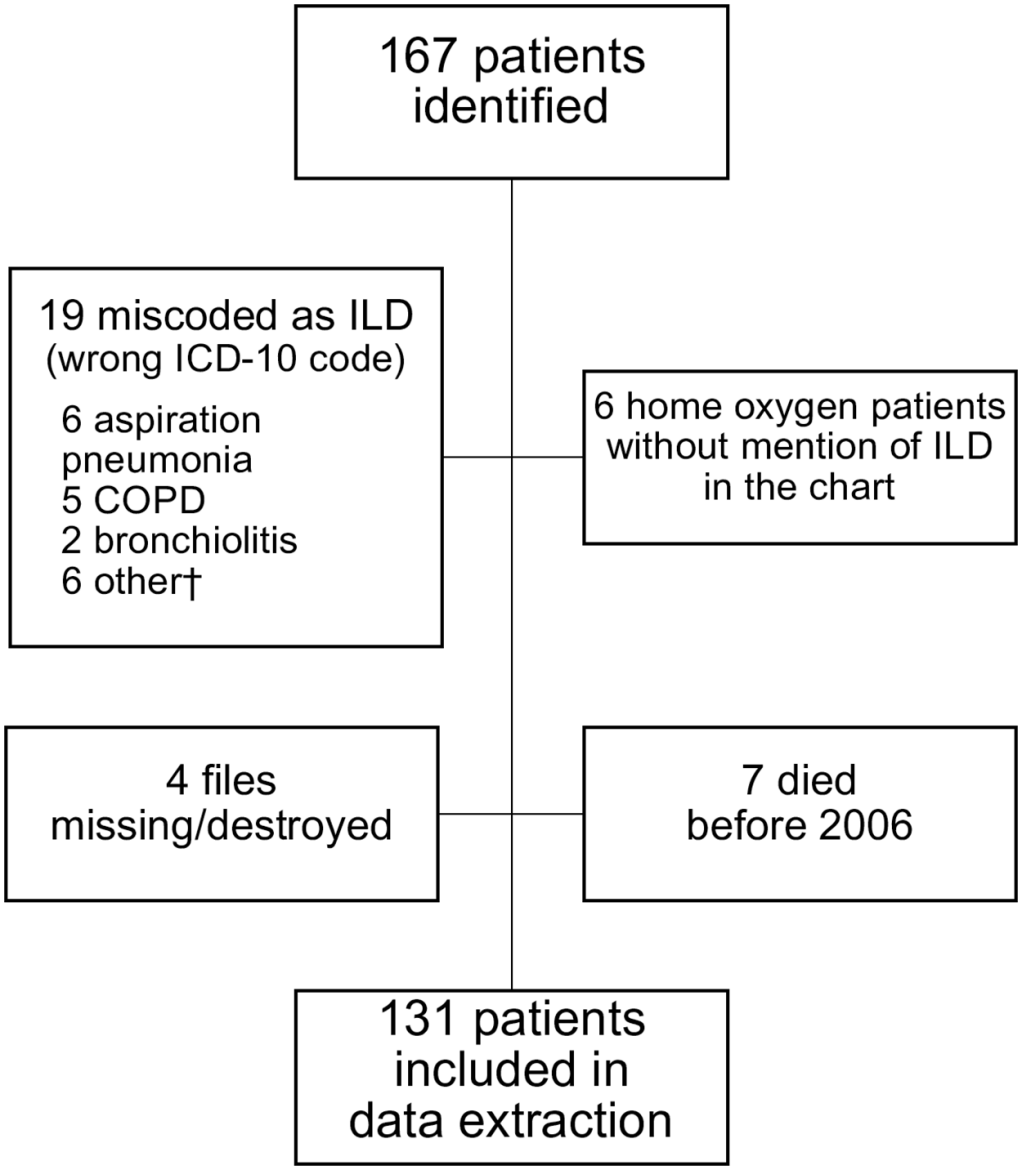
Reasons for exclusion of potential cases from the detailed data extraction. Abbreviations: COPD–chronic obstructive pulmonary disease, ILD–interstitial lung disease. †Other: congestive heart failure, drowning, cancer, toxic inhalation, pneumonia.

There were 167 potential cases of ILD identified. Of these, 36 were excluded from further data extraction (see Fig 2 for details) because it was determined that the incorrect ICD-10 code had been used (n = 19), or they had been identified as a home-oxygen user but no mention of ILD was found in their medical records (n = 6), their medical records were missing (n = 4), or they had died prior to 2006 (n = 7). The remaining 131 had data extracted and reviewed.

### Categorization as Definite ILD

**Fig 3:**
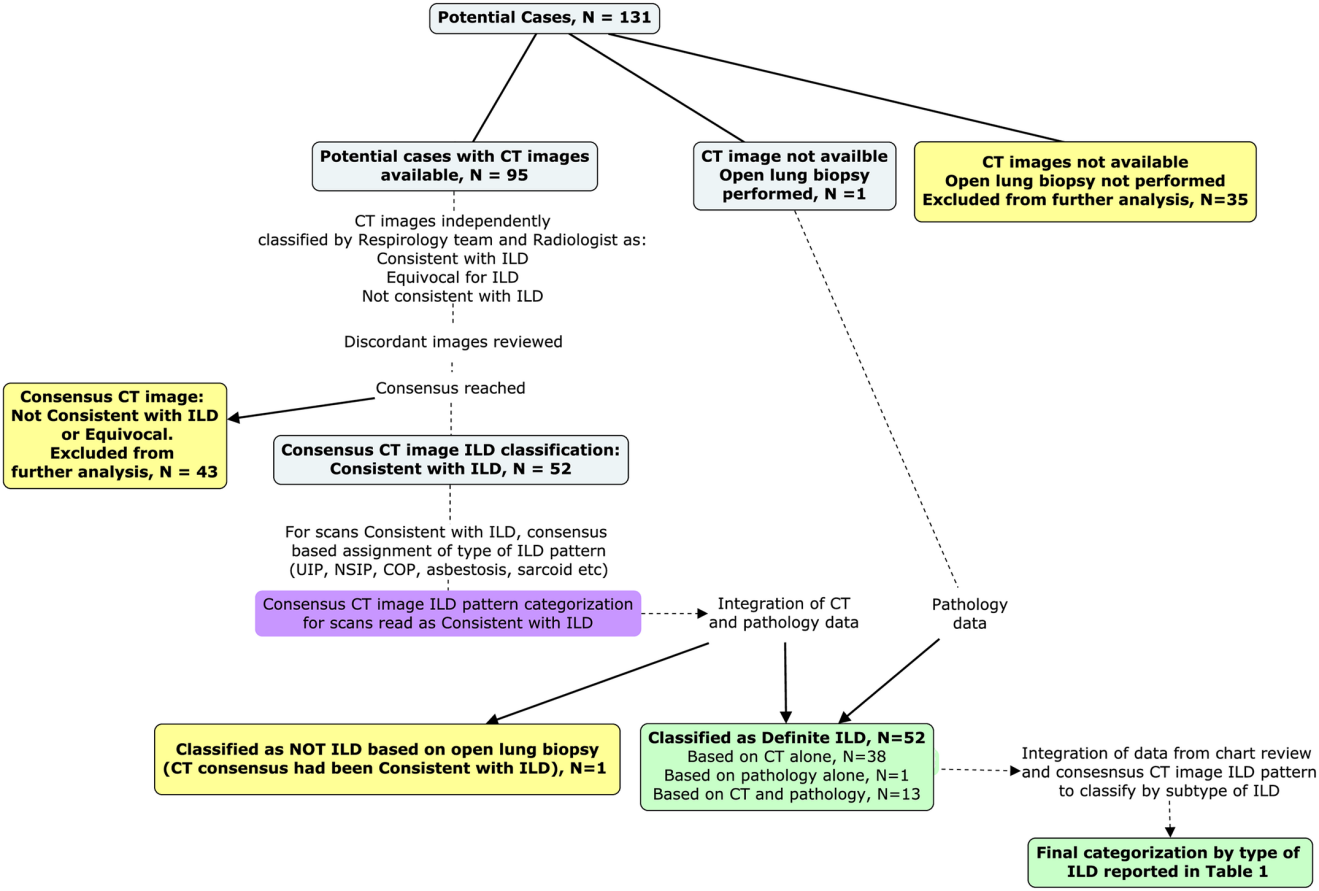
Process for classification of potential cases as Definite ILD and by subtype of ILD. Figure was created using CMAP Tools v 6.00.4 (University of West Florida.Institute for Human and Machine Cognition. (2000). IHMC CmapTools, Institute for Human and Machine Cognition.) Blue boxes indicate patients not yet classified. Yellow boxes indicate where potential cases were excluded from classification as Definite ILD. Green boxes indicate patients classified as Definite ILD.

Classification of the 131 potential cases is depicted in Fig 3. Of 131 potential cases, 35 (27%) did not have CT scan imaging available for review and had not undergone open lung biopsy. These potential cases were ineligible for being classified as Definite ILD.

Thoracic CT imaging was available for review in 95/131 (73%) potential cases, with a median of 3 scans per potential case (range 1–12). CT imaging was classified as consistent with ILD in 52/95 (55%) potential cases; and as not consistent with ILD in 38/95 (40%). In the remaining 5/95 (5%), CT images were considered equivocal for ILD. The weighted kappa statistic for agreement between the Respirology and Radiology interpretation was 0.71 (95%CI: 0.61–0.82). Fifteen potential cases had undergone open lung biopsy (15/131, [11%]); amongst these, ILD was found on histopathologic examination in 14/15 (93%) and an alternative diagnosis was made (lymphangitic carcinomatosis) in 1/15 (7%). Upon integration of histopathology data, we found that CT imaging had been classified as consistent with ILD in all 14 cases of biopsy-proven ILD and in one non-ILD case.

Overall, criteria for Definite ILD were met in 52/131 (40%) potential cases (14 on the basis of lung biopsy, 38 on the basis of imaging alone).

### Categorization by subtype of ILD

In 14/52 cases of Definite ILD, we used histopathology reports to determine the subtype of disease present, which were: usual interstitial pneumonia (n = 9); unclassifiable fibrosis in (n = 3); NSIP (n = 1); and hypersensitivity pneumonitis (n = 1). For the 38/52 in whom open lung biopsy had not been performed, the following ILD subtype pattern classifications were made by reviewing CT images: usual interstitial pneumonia (n = 23); NSIP (n = 13); sarcoidosis (n = 1); and asbestosis (n = 1).

The chart review identified six patients as having a past medical history of connective tissue disease (n = 4 rheumatoid arthritis, n = 1 scleroderma, n = 1 mixed connective tissue disease); these were classified as either secondary UIP (5 of 32 with UIP), or secondary NSIP (1 of 14 with NSIP). Occupational history had not been recorded in the majority of the Definite ILD patients’ charts (missing in 34/52). For the 18 cases where some occupational history was available, data were quite limited and lacked details on duration and exposures. The occupations found in the chart review are listed in S2 Table; for two patients that had worked as miners, CT images were not compatible with pneumonoconiosis; for six patients that had been employed as maintenance or construction workers, scans were not consistent with prior asbestos exposure or with asbestosis in 5 of 6, and consistent with asbestosis in 1.

The breakdown of the final categorization by subtype of ILD was: IPF (27/52, 52%); idiopathic NSIP (13/52, 25%); secondary usual interstitial pneumonia associated with connective tissue disease (5/52, 10%); “unclassifiable fibrosis” (3/52, 6%); NSIP associated with connective tissue disease (1/52, 2%); sarcoidosis (1/52, 2%); hypersensitivity pneumonitis (1/52, 2%); and asbestosis (1/52, 2%).

### Patient characteristics and survival

Patient characteristics are summarized in Table 1, for Definite ILD and each subtype. Most patients were diagnosed in their seventh decade of life or later, and age distributions were similar across the four most common subtypes. While both sexes were equally affected by ILD overall, discrepancies were seen for specific subtypes: women accounted for the minority of IPF (37%), and the majority of the other three most common subtypes. Tobacco smoking was prevalent overall (77%), with the highest proportion of smokers in the IPF and secondary usual interstitial pneumonia subtypes. Across all subtypes, the majority of participants did not have pulmonary function data available within one year of their date of diagnosis; in cases where these data were available, both forced vital capacity and diffusing capacity were suggestive of advanced disease at the time of diagnosis.

**Table 1:** Characteristics of Eeyou Istchee residents with interstitial lung disease (‘Definite ILD’), overall and stratified by subtype.

Variable	All with Definite ILD	IPF	Idiopathic NSIP	Secondary UIP	Un-classifiable fibrosis*	Hyper-sensitivity pneumonitis*	Secondary NSIP^†^	Sarcoid^†^	Asbestosis^†^
n, (% of those with Definite ILD)	52 (100%)	27 (52%)	13 (25%)	5 (10%)	3 (6%)	1 (2%)	1 (2%)	1 (2%)	1 (2%)
Year of diagnosis, median (IQR)	2008 (2006,2010)	2009 (2007,2010)	2008 (2004,2010)	2008 (2007,2009)	2010 (2002,2012)	2007	2006	2006	1990
Age of diagnosis, median (IQR)	66 (57–70)	63 (56–71)	67 (63–74)	66 (62–68)	67 (47–70)	51	41	40	59
Female	27 (52%)	10 (37%)	9 (69%)	4 (80%)	2 (67%)	1 (100%)	1 (100%)	0	0
Ever smoker	36 (77%)	22 (88%)	5 (50%)	5 (100%)	1 (33%)	0	1 (100%)	1 (100%)	1 (100%)
% predicted FVC within one year of diagnosis, median (IQR)	55 (41–66)	55 (40–66)	44 (39–73)	80 (61–100)	58 (54–61)	53	no data	50	
% predicted D_L_CO within one year of diagnosis, median (IQR)	45 (34–56)	42 (27–57)	66 (55–76)	49 (40–61)	36 (33–39)	40	no data	no data	
Died	27 (52%)	16 (59%)	5 (38%)	4 (80%)	2 (67%)	0	0	0	0
Median survival from date of diagnosis, years (95%CI)	4.0 (2.4–6.5)	4.8 (1.7–6.5)	Inestimable	2.4 (0.7–4.0)	3.1 (2.9–3.4)	N.A.	N.A.	N.A.	N.A.

Both prevalent and incident cases are included in the table. ILD, interstitial lung disease; IPF, idiopathic pulmonary fibrosis; NSIP, non-specific interstitial pneumonia; UIP, usual interstitial pneumonia; IQR, interquartile range

* Diagnosis based on open lung biopsy.

^†^ Diagnosis of NSIP based on review of CT imaging, classification as secondary based on past medical history of connective tissue disease.

Data missing for Ever smoker: Definite ILD, n = 5; IPF, n = 2; idiopathic NSIP, n = 3. Data missing for FVC: Definite ILD, n = 29; IPF, n = 16; idiopathic NSIP, n = 9; secondary UIP, n = 1; unclassifiable fibrosis, n = 1. Data missing for DLCO: Definite ILD, n = 36; IPF, n = 20; idiopathic NSIP, n = 11; secondary UIP, n = 1; unclassifiable fibrosis, n = 1

**Fig 4:**
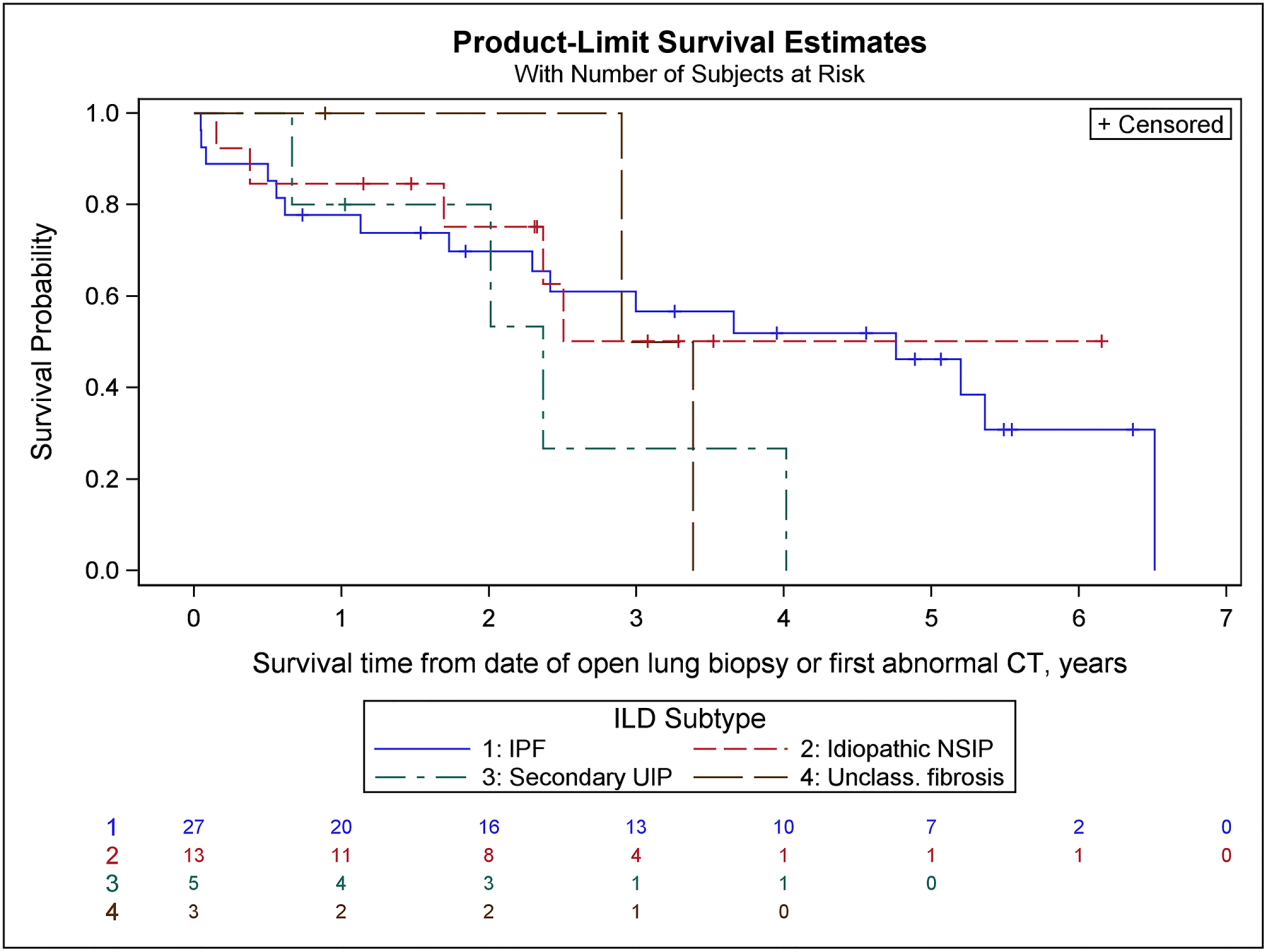
Survival curves for the four most common interstitial lung disease (ILD) subtypes. Numbers in rows at bottom of figure are the number surviving. IPF: idiopathic pulmonary fibrosis; NSIP: non-specific interstitial pneumonitis; UIP: usual interstitial pneumonia; Unclass. fibrosis: unclassifiable fibrosis.

Survival curves for the four most common subtypes are shown in Fig 4. Survival was poorest for secondary usual interstitial pneumonia, followed by unclassifiable fibrosis and IPF—but number of cases was small in all groups, with the exception of the IPF group.

### Incidence of ILD

Excluding 11 patients diagnosed with ILD before January 1^st^ 2006, there were 41 incident cases during our study period. Amongst these, 23 had IPF, and 8 had idiopathic non-specific interstitial pneumonia.

As shown in Table 2, the crude annual incidence of ILD was 32 per 100,000 PYO, and the age-standardized incidence was 80 per 100,000 PYO, between January 1^st^ 2006 and December 31^st^ 2013. For IPF, the crude and age-standardized incidence rates were 18 and 46 per 100,000 PYO, respectively.

**Table 2:** Crude and age-standardized annual incidence of ILD and IPF among residents of Eeyou Istchee, Jan 1^st^ 2006- Dec 31^st^ 2013. Rates are per 100,000 PYO.

	Number of incident cases	Crude rate	Age standardized rate (95%CI)
**ILD**	41	32	80 (55–106)
**IPF**	23	18	46 (26–65)

Abbreviations: ILD: interstitial lung disease, IPF: idiopathic pulmonary fibrosis, PYO: person-years observed

## Discussion

To our knowledge, ours is the first epidemiologic investigation of ILD in an Indigenous population. We found that the age adjusted annual incidence of ILD in Eeyou Istchee between January 1^st^ 2006 and December 31^st^ 2013 was 80 per 100,000 PYO; and for IPF, the most common subtype, was 46 per 100,000 PYO. Our estimated incidence of Definite ILD in Eeyou Istchee is 2.3-fold higher than the incidence that was estimated for the province of Quebec (35 per 100,000 PYO) in a recently published study by another group of investigators.[16] The province-wide incidence was estimated using healthcare administrative data without validating diagnoses through reviewing images and histopathology. In our study, we would have grossly overestimated the incidence of ILD had we not reviewed data to validate diagnoses, as 79/131 (60%) of potential cases did not meet criteria for Definite ILD. The importance of validating ILD diagnoses when using healthcare databases to study ILD epidemiology has also been shown by investigators in Finland who estimated that the absence of clinical review would have resulted in overestimating the number of cases by 23–51%.[17,18] Hence, it is likely that directly comparing our estimated incidence of ILD with the province-wide incidence that was estimated[16] without case verification, will underestimate the excess occurrence of this group of diseases in Eeyou Istchee.

In our study, IPF was the most common ILD. In a recent systematic review, the average IPF incidence in Europe and North America was estimated to be 2.8 to 9.3 per 100,000 person years (after exclusion of studies that could have been potential outliers).[19] As shown in Table 3, the crude incidence of IPF is higher in Eeyou Istchee compared to estimates in other North American and European populations from studies that validated diagnoses.[8,19,20] However, the Eeyou Istchee population’s age distribution differs from most North American and European countries as it is skewed towards the younger age groups, which is typical of Canada’s Indigenous populations. Therefore, it is more appropriate to use the age-adjusted incidence when comparing with that of mostly non-Indigenous populations. The age-adjusted incidence of IPF in Eeyou Istchee is higher than the incidence reported in all but one of the studies included in the recent systematic review of IPF epidemiology;[19] moreover, the one study that did estimate a higher incidence was restricted to persons at least 65 years old,[11] an age group at higher risk of IPF. The main difference between our study and others is that we supplemented the search of health care administrative data by conducting a survey of physicians and a review of lists of patients on home oxygen treatment at local clinics. However, if we exclude patients identified by these additional methods when estimating the incidence of ILD and IPF, the rates in Eeyou Istchee are still much higher than reported elsewhere: for ILD, 71 (47–95) per 100,000; and for IPF 59 (37–81) per 100,000. These rates are even more striking when one considers that they are likely lower-bound estimates of the true incidence in this region because 27% of potential cases had neither CT imaging or histopathology data available, and as a result, were not eligible for classification as Definite ILD.

**Table 3:** Comparison of the annual incidence of IPF per 100,000 PYO in Eeyou Istchee to the incidence of IPF in other populations from studies that used similar methods of case identification and diagnostic verification.

Geographic location	Crude rate	Age-adjusted rate
**Eeyou Istchee**	18	46
**Minnesota, USA** [19,20]	Not reported	8.8
**New Mexico, USA** [8,19]	10.7 (males) 3.4 (females)	Not reported
**Europe** [19]	1.4–9.3	Not reported

The median survival estimated for IPF patients in our study was 4.8 years, which is similar to that reported in other recent studies of IPF. For example, Strand et al reported a median survival of 4.4 years in a cohort of 321 IPF patients diagnosed in Denver, Colorado;[21] and Hopkins et al reported 59% of 1151 incident IPF cases were alive 4 years after diagnosis in Ontario, Canada.[22] The longer survival in our cohort, and also in the study by Hopkins et al, might be because we measured survival time from dates other than the date of open lung biopsy for some patients, whereas other studies strictly used the date of open lung biopsy. The longer survival in our study may also indicate some degree of misclassification of the type of ILD i.e. if we erroneously classified patients as having IPF when they truly had another ILD with a better prognosis. However, our estimated median survival is overall quite similar to that reported in other studies, which argues against substantial misclassification. A number of studies have reported longer survival for patients with usual interstitial pneumonia secondary to connective tissue diseases compared to those with IPF, with the exception of patients with undifferentiated connective tissue disease.[21,23–25] In our study, prognosis was worst for patients with usual interstitial pneumonia associated with connective tissue disease, their median survival was only 2.4 years. While we hesitate to over-interpret this estimate given there were only five individuals in this category, their shorter survival could indicate methodological limitations with regards to the identification of the presence or type of connective tissue disease (e.g. these may have been erroneously recorded in the medical charts of some patients), or with regards to our classification, i.e. the shorter survival could indicate that this group may, in fact, have had IPF and we were incorrect in attributing their ILD to the connective tissue disease.

### Strengths and limitations of the study

Amongst the strengths of our study are our rigorous search for potential cases, thorough validation of diagnoses, and strict criteria for being classified as Definite ILD, which contributed to ensuring that our estimates were accurate. Additionally, the availability of detailed data on population age distribution, for Eeyou Istchee and the rest of the province, allowed us to calculate age-adjusted incidence rates, which were essential for demonstrating the elevated occurrence of ILD and IPF in Eeyou Istchee.

Our study also has limitations. First, as mentioned above, it is likely that we underestimated the incidence because 27% of potential cases were missing CT images and histopathologic data. Second, most Definite ILD cases had not undergone open lung biopsy—however, this is unlikely to affect the validity of our findings given the categorization was based on a rigorous review of CT images; moreover, the most common radiologic pattern identified was usual intersitital pneumonia, a radiologic finding that is strongly predictive of the same pattern on histolopathologic examination.[14,26,27] Third, our reporting of patient characteristics was limited because we relied on data collected as part of routine clinical care which were often incomplete or missing. Fourth, the quality of CT scans varied over time and between healthcare institutions, which may have resulted in some misclassification particularly of the ILD pattern. Lastly, our reliance on information recorded in medical records meant that data for accurately differentiating between primary and secondary causes of ILD were often missing, as were data on family histories that could have helped to assess the possibility of genetic susceptibility in this population.

## Conclusion

Our findings suggest that the incidence of ILD, and particularly of IPF, are higher in Eeyou Istchee than in other populations in Quebec and elsewhere. It is important to interpret our estimates with caution due to the lack of histopathological confirmation in the majority of cases, and our reliance on chart review to exclude secondary causes. A prospective study of incident cases with standardized assessments to establish the types of ILD and to assess for potential causes could overcome some of the limitations of the present analysis. Additional research is warranted to elucidate the causes of the seemingly elevated incidence, to assess quality of life of ILD patients, and identify strategies to optimize diagnosis and management in this region. Studies evaluating ILD and IPF incidence in other Indigenous populations would also be of interest.

## Supporting information

S1 Table List of ICD-10 codes provided to hospital archives to identify patients that carried a diagnosis of ILD. (DOCX)

S2 Table Data on occupation obtained from chart review. (DOCX)
