# Supplementary material for: Estimating the incidence of interstitial lung diseases in the Cree of Eeyou Istchee, northern Québec
Source: PLoS One. 2017 Sep 8;12(9):e0184548. doi: 10.1371/journal.pone.0184548 (PMC5590969; doi:10.1371/journal.pone.0184548)
Supplement: S1 Table — (DOCX) [file pone.0184548.s001.docx]

**Supplement to:**

**Storme, M et al. “Estimating the incidence of interstitial lung diseases in the Cree of Eeyou Istchee, northern Québec”**

Appendix

S1 Table. List of ICD-10 codes provided to hospital archives to identify patients that carried a diagnosis of ILD.

| **ICD-10 code** | **Description** |
| --- | --- |

| **J60** | Coal worker pneumoconiosis |
| --- | --- |
| **J61** | Pneumoconiosis due to asbestos and other mineral fibers |
| **J62** | Pneumoconiosis due to dust containing silica |
| **J63** | Pneumoconiosis due to other inorganic dusts |
| **J64** | Unspecified pneumoconiosis |
| **J67** | Hypersensitivity pneumonitis due to organic dust |
| **J68** | Respiratory conditions due to inhalation of chemicals, gases, fumes and vapors |
| **J70** | Respiratory conditions due to other external agents |
| **J84** | Other interstitial pulmonary diseases |
| **B22.1** | HIV disease resulting in lymphoid interstitial pneumonitis |
| **D86** | Sarcoidosis |
| **J99.0** | Rheumatoid lung disease |
| **J99.1** | Respiratory disorders in other diffuse connective tissue disorders |
| **T59** | Toxic effects of other gases, fumes and vapors |
