# Supplementary material for: Estimating the incidence of interstitial lung diseases in the Cree of Eeyou Istchee, northern Québec
Source: PLoS One. 2017 Sep 8;12(9):e0184548. doi: 10.1371/journal.pone.0184548 (PMC5590969; doi:10.1371/journal.pone.0184548)
Supplement: S2 Table — (DOCX) [file pone.0184548.s002.docx]

**Supplement to:**

**Storme, M et al. “Estimating the incidence of interstitial lung diseases in the Cree of Eeyou Istchee, northern Québec”**

Appendix

**S2 Table. Data on occupation from chart review.**

| Subtype of Definite ILD | Occupation identified in chart review |
| --- | --- |
| IPF | Manager |
| IPF | Salesperson |
| IPF | Security guard |
| IPF | Maintenance worker |
| IPF | Maintenance worker, soapstone carver |
| IPF | Construction worker, uranium miner |
| IPF | Hunter |
| IPF | Construction worker |
| IPF | Welfare agent |
| IPF | Artist |
| IPF | Security guard, miner, lumberjack, fireman |
| IPF | Hunter |
| Idiopathic NSIP | Hunter |
| Secondary UIP | Maintenance worker |
| Secondary UIP | Hunter |
| Unclassifiable fibrosis | Hunter, carpenter |
| Hypersensitivity pneumonitis | Social worker |
| Asbestosis | Retired, construction worker, known asbestos exposure |
